# Transcriptional Responses of Escherichia coli to a Small-Molecule Inhibitor of LolCDE, an Essential Component of the Lipoprotein Transport Pathway

**DOI:** 10.1128/JB.00502-16

**Published:** 2016-11-04

**Authors:** Christian Lorenz, Thomas J. Dougherty, Stephen Lory

**Affiliations:** Department of Microbiology and Immunobiology, Harvard Medical School, Boston, Massachusetts, USA; University of Wisconsin—Madison

## Abstract

In Gram-negative bacteria, a dedicated machinery consisting of LolABCDE components targets lipoproteins to the outer membrane. We used a previously identified small-molecule inhibitor of the LolCDE complex of Escherichia coli to assess the global transcriptional consequences of interference with lipoprotein transport. Exposure of E. coli to the LolCDE inhibitor at concentrations leading to minimal and significant growth inhibition, followed by transcriptome sequencing, identified a small group of genes whose transcript levels were decreased and a larger group whose mRNA levels increased 10- to 100-fold compared to those of untreated cells. The majority of the genes whose mRNA concentrations were reduced were part of the flagellar assembly pathway, which contains an essential lipoprotein component. Most of the genes whose transcript levels were elevated encode proteins involved in selected cell stress pathways. Many of these genes are involved with envelope stress responses induced by the mislocalization of outer membrane lipoproteins. Although several of the genes whose RNAs were induced have previously been shown to be associated with the general perturbation of the cell envelope by antibiotics, a small subset was affected only by LolCDE inhibition. Findings from this work suggest that the efficiency of the Lol system function may be coupled to a specific monitoring system, which could be exploited in the development of reporter constructs suitable for use for screening for additional inhibitors of lipoprotein trafficking.

**IMPORTANCE** Inhibition of the lipoprotein transport pathway leads to E. coli death and subsequent lysis. Early significant changes in the levels of RNA for a subset of genes identified to be associated with some periplasmic and envelope stress responses were observed. Together these findings suggest that disruption of this key pathway can have a severe impact on balanced outer membrane synthesis sufficient to affect viability.

## INTRODUCTION

Multiply drug-resistant (MDR) bacterial pathogens pose a serious challenge in clinical medicine. Currently, the options for the treatment of serious infections caused by Gram-negative organisms are narrowing. With the emergence of carbapenem-resistant Enterobacteriaceae (CRE), it is clear that new sources of efficacious compounds to address infections caused by Gram-negative bacteria are a necessity (1, 2). The presence of two dissimilar membranes surrounding Gram-negative bacteria, a cytoplasmic membrane and a outer membrane, presents a particular challenge to antibiotic therapy of infections caused by this group of organisms (3–5). Whereas the inner cytoplasmic membrane has properties of a typical lipid bilayer, the outer membrane has an asymmetric character, with a phospholipid-containing inner surface and an outer surface consisting largely of lipopolysaccharide. Proteins of the inner membrane are mostly typical membrane proteins with α-helices and transmembrane loops, whereas the majority of outer membrane proteins have either β-barrel structures or are lipoproteins (4, 6). Various small molecules, including nutrients or antibiotics, that need to reach the cytoplasm often penetrate the outer membrane by diffusion through the hydrophilic channels of β-barrel porins. The antibiotics subsequently traverse the inner membrane primarily through diffusion across the phospholipid bilayer, requiring some degree of lipophilicity and a neutralized charge (zwitterionic properties) (7). Because of the membranes' orthogonal properties, it has been difficult to identify antibiotics that have the chemical properties needed to penetrate both the outer and inner membranes (7, 8). An additional challenge to the effective eradication of Gram-negative bacteria is the presence of broad-substrate efflux pumps in the periplasm which act to reduce antibiotic concentrations in the bacteria (9).

The unique components of the outer membrane of Gram-negative bacteria are assembled during cell elongation and division. Three outer membrane assembly pathways with components located in each of the membranes and in the periplasm are known to exist in these bacteria: Bam (β-barrel assembly machine), Lpt (lipopolysaccharide transport proteins), and Lol (lipoprotein transport) (10–12). Each of these is essential for the biogenesis of a functional outer membrane. Compromising the structure of the outer membrane not only could potentially lead to improved kinetics of penetration of existing antibiotics into Gram-negative bacterial pathogens but also could disrupt the assembly or function of the tripartite efflux pumps.

In Escherichia coli there are more than 90 different lipoproteins, with the majority residing in the inner leaflet of the outer membrane (12). The components of the Bam, Lpt, and Lol pathways include essential lipoproteins; therefore, disruption of lipoprotein synthesis leads to an imbalance in outer membrane biogenesis caused by a malfunction in all three systems (12). The lipoprotein transport pathway has five protein components: the LolCDE complex provides the energy for transport, is essential, and resides in the cytoplasmic membrane, while LolA is localized in the periplasm and LolB is an outer membrane lipoprotein (13). The LolCDE complex of E. coli has been shown to consist of one copy each of the membrane-spanning subunits LolC and LolE and two copies of the ATPase subunit LolD (14). On the basis of the current model for lipoprotein transport in E. coli and likely in all Gram-negative bacteria, the lipoprotein precursors are acylated on the sulfhydryl of the cysteine in a consensus lipobox sequence, and following the cleaved signal peptide, the newly created N terminus of the cysteine is also acylated. These reactions are carried out sequentially in the inner membrane by three enzymes, Lgt, LspA, and Lnt (12). Following these covalent modifications, LolCDE catalyzes the release of the lipoproteins destined for the outer membrane from the inner membrane to the periplasmic lipoprotein carrier, LolA (15, 16). LolA in turn transports the lipoprotein across the periplasm to the outer membrane, where LolB accepts the nascent lipoproteins and facilitates their insertion into the outer membrane (17).

**FIG 1:**
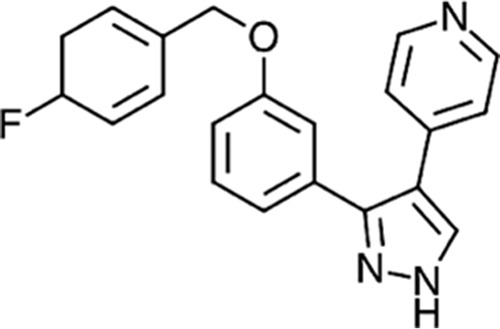
Chemical structure of the LolCDE inhibitor compound 2. (Republished from reference 19.) The compound has a molecular weight of 345.4 and a measured distribution coefficient (log *D*) of 4.3.

Until recently, the only known small molecule capable of interfering with lipoprotein transport was globomycin, an inhibitor of the type II signal peptidase Lsp (18). Employing a general cell wall reporter assay, Nayar et al. reported the discovery of a small-molecule inhibitor of the Lol pathway in E. coli (19). This molecule contained a pyrazole core and had a molecular weight of 345.4 (Fig. 1). It exhibited potent activity against efflux-deficient E. coli with a MIC of 0.125 to 0.25 μg/ml. This compound was shown to inhibit Lol transport by demonstration of the blocking of the release of Lpp from E. coli spheroplasts and by isolation of resistant mutants with gene mutations leading to amino acid changes in either the LolC or LolE component of the lipoprotein transport machinery. Since these two proteins show modest sequence conservation (27% identity), it is conceivable that the compound recognizes a structurally related fold interfering with the function or assembly of the LolCDE complex.

We exploited the activity of the small-molecule inhibitor of the Lol pathway to investigate additional physiological effects of interference with lipoprotein transport in E. coli. The impact of the compound on bacterial viability and cell integrity over time was assessed, and the early transcriptional changes brought about by exposure to the compound are described. In addition, we compared the transcriptional levels of several key genes with altered expression levels resulting from inhibition of the Lol pathway with the transcriptional levels in the presence of several antibiotics with different mechanisms of action. Our findings demonstrate that pharmacological inhibition of the Lol pathway results in increased transcription in several envelope stress pathways.

## MATERIALS AND METHODS

### Bacterial strains and growth.

For all experiments, Escherichia coli BW25113 [*rrnB3* Δ*lacZ4787 hsdR514* Δ(*araBAD*)*567* Δ(*rhaBAD*)*568 rph-1*] with the Δ*acrB* deletion (CGSC JW0451-2 8609) was employed (20). Use of the Δ*acrB* strain allowed the use of lower concentrations of the limited supply of the LolCDE inhibitor compound (compound 2), for which the MIC in LB is 0.6 μg/ml. All experiments were performed in LB broth cultures at 37°C with shaking at 300 rpm. For experiments in which compounds or antibiotics were added, the MIC values determined in LB broth microtiter plates with low concentrations of bacterial cells (5 × 10^5^ CFU/ml) (21) were used as guidelines to test bacterial growth at the higher cell densities needed for the RNA extractions. Cells were grown in larger cultures, and compound or antibiotic was added at concentrations below and above the broth microdilution MICs. Growth was monitored using the optical density at 600 nm (OD_600_), and the concentrations that produced reductions in the growth rate were employed for the experiments (see Fig. S1 in the supplemental material).

Resistant mutants of BW25113 Δ*acrB* were selected on LB agar plates with 5 μg/ml (8× MIC) of compound 2. After 20 h of incubation, selected colonies were transferred onto fresh LB plates with 5 μg/ml of compound 2 and grown overnight at 37°C. The growth from these plates was stored in 10% glycerol in LB at −80°C. Genomic DNA was isolated from several clones, and LolC and LolE were amplified. A mutant with a previously reported (19) mutation in LolC (N256K) that confers resistance to the compound was selected for further study. The MIC for this clone was measured in LB with serial dilutions of compound. The MIC increased from 0.6 μg/ml for the parent to 32 μg/ml for the mutant.

### Compound.

Compound 2 (19) was obtained in a powder form from AstraZeneca, Waltham, MA. It was dissolved in dimethyl sulfoxide to obtain a 5-mg/ml stock and kept at −20°C. The MIC for E. coli BW25113 was periodically checked to ensure the retention of activity.

### Cell-killing kinetics.

The rate of growth/killing of E. coli BW25113 Δ*acrB* was determined by treating mid-logarithmic-phase cells with a range of increasing concentrations of the LolCDE inhibitor. Samples were removed at timed intervals and serially diluted in 10-fold series, and 100-μl aliquots from each dilution were spread on plates containing LB agar. The plates were incubated for 24 h, and cells were enumerated by counting the colonies. Bacterial lysis was monitored by following the changes in the OD_600_, after periodically taking samples, diluting them into the linear range, and measuring the absorbance on a VWR UV 1600 PC spectrophotometer.

### Transcriptome analysis by RNA-seq.

For transcriptome sequencing (RNA-seq), E. coli BW25113 Δ*acrB* was grown overnight in LB broth with shaking at 37°C. On the next morning, a 1:200 dilution was made in 150 ml of LB, and the bacteria were grown at 37°C with shaking until an OD_600_ of 0.5 was attained. The culture was then split into six portions of 20 ml each that were placed into six flasks, with two flasks being used as biological replicate controls, two replicate flasks receiving 0.3 μg/ml of the LolCDE inhibitor, and two replicate flasks receiving 1.2 μg/ml of the LolCDE inhibitor. After 30 min, 800 μl of culture from each flask was placed directly into prewarmed (65°C) lysis mix-acid phenol solution. Lysis mix consisted of 320 mM sodium acetate, 8% SDS, and 16 mM EDTA (all from Ambion, Thermo Fischer) in nuclease-free water. One hundred microliters of the above-described lysis mix was combined with 700 μl of acid phenol-chloroform (Ambion) in 2-ml tubes. The cells and lysis mix-acid phenol were rapidly mixed on a vortex mixer and kept at 65°C with vortexing for 5 to 10 s every minute for 10 min. After centrifugation for 5 min at 12,000 × *g*, the upper phase was carefully removed, transferred into 700 μl of phenol-chloroform-isoamyl alcohol (Ambion), and vortexed to mix. This extraction procedure was repeated twice, and centrifugation at 12,000 × *g* for 5 min was used to separate the phases, with the upper phase being taken each time. Finally, the upper phase was transferred to 600 μl (equal volume) of chloroform-isoamyl alcohol and again centrifuged as described above. The upper phase was removed, and RNA was precipitated by treatment with 2 volumes of 100% ethanol overnight at 80°C. On the following morning, the mixture was centrifuged at 18,000 × *g* at 4°C for 10 min. The pellets were washed once with 70% ethanol in DNase- and RNase-free water (Invitrogen). The pellets were dried under vacuum in a Savant SpeedVac system for 5 to 10 min. The dried pellets were resuspended in 30 μl of diethyl pyrocarbonate-treated water (Invitrogen). RNA concentrations were determined using a NanoDrop spectrophotometer (Thermo Fisher). Ribosome integrity numbers (RINs) were determined with an Agilent Bioanalyzer 2100 instrument and an Agilent RNA 6000 Nano kit (Santa Clara, CA). The initial RIN for all six RNA samples was 10. DNase treatment was carried out with a Turbo DNA-free kit (Ambion). The RINs were again checked after DNase treatment, and the values ranged from 9.4 to 9.9. The rRNA was depleted using a Ribo Zero rRNA removal kit for Gram-negative bacteria (Illumina). RNA libraries were prepared with an NEBNext ultradirectional RNA library preparation kit for the Illumina system using NEBNext multiplex oligonucleotides for Illumina index primer set 1 (New England BioLabs). The size distribution of the library was tested with an Agilent 2200 TapeStation high-sensitivity D1000 ScreenTape system. RNA-seq was carried out on an Illumina HiSeq platform, with the coverage of the six samples ranging from 18 million to 24 million reads each. Analysis of the data was performed using CLC Bio Genomics Workbench software, with the reads being mapped to the genome sequence of E. coli BW25113. Duplicate sample data were averaged, and the complete comparative data for RNA-seq are provided in Tables S1 and S2 in the supplemental material. Low-level expression data were eliminated using the formula for expression cutoff in terms of the number of reads per million (22).

### qPCR.

For determination of RNA levels by quantitative PCR (qPCR), various concentrations of antibiotic were first tested in cultures grown in 20-ml volumes. The concentrations of antibiotics that were twice the lowest level required to inhibit growth, determined by measurement of the optical density (see Fig. S1 in the supplemental material), were selected, and in a subsequent experiment cells were incubated with those concentrations for 30 min. RNA was then prepared from these cultures with the hot acid phenol procedure as described above. Primers were designed by use of the GenScript real-time PCR primer design tool. cDNA was synthesized with a SuperScript III first-strand synthesis system for reverse transcription-PCR (Invitrogen) and random hexamer primers. The qPCR was carried out using PerfeCTa SYBR Green FastMix (Quanta Biosciences) in a Mastercycler Realplex^2^ system from Eppendorf. Changes in transcript levels relative to the levels in the untreated control cultures were calculated. The growth and exposure for RNA extractions for the qPCR experiments with 30-min compound exposures were performed twice (biological replicates) on different days.

## RESULTS

### Effect of Lol inhibition on growth.

**FIG 2:**
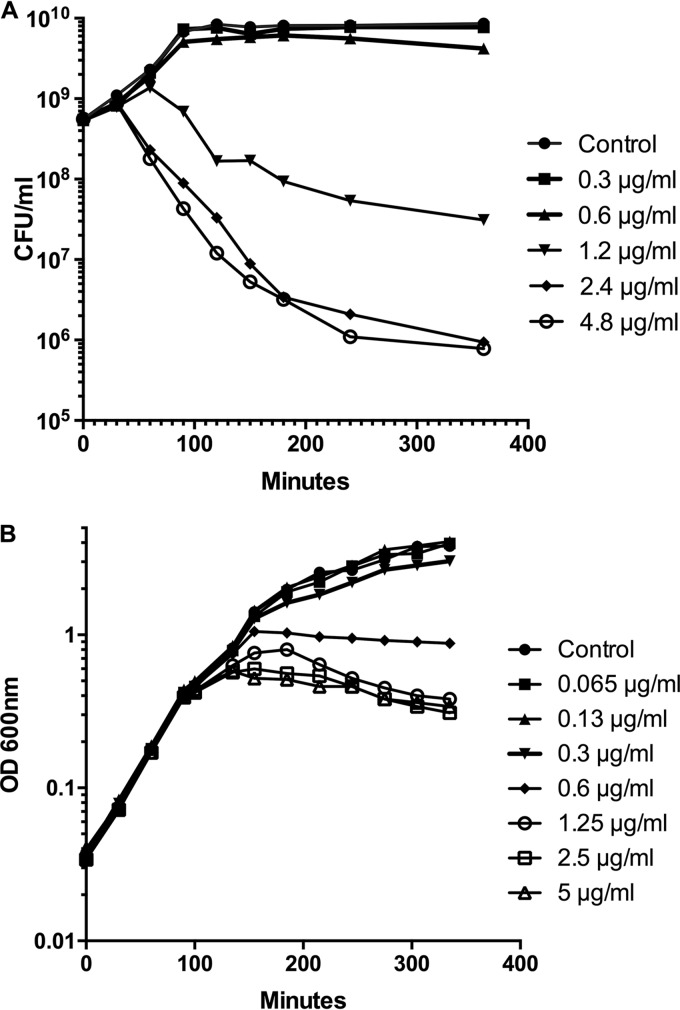
(A) Cell killing by the LolCDE inhibitor. E. coli BW25113 Δ*acrB* cells from an overnight culture were inoculated and grown to an OD_600_ of 0.5. The indicated concentrations of compound were added, and duplicate samples were periodically taken from each culture. The samples were serially diluted 10-fold, and aliquots of each replicate were plated in duplicate on LB agar plates (2 replicate samples each were diluted on 2 plates). After incubation, the colonies on plates with between 20 and 250 colonies were counted. The average of the four plate counts was calculated and plotted. (B) Measurements of the optical density at 600 nm versus time in the presence of the LolCDE inhibitor. The optical densities of the cultures that were used as a source of RNA for RNA-seq were determined before and after the 30-min exposure time to observe the longer-term effects of the compound on cell growth. The indicated broad range of concentrations of the compound was added when the cultures reached an OD_600_ of 0.5. After 30 min of exposure to the compound at 0.3 and 1.2 μg/ml, samples for determination of RNA levels were removed from the control culture and the cultures were treated with the compound.

Previous work has established that the inhibitor (compound 2) (Fig. 1) (19) blocks lipoprotein transport and that resistance to the compound is found in bacterial cells with amino acid substitutions in either LolC or LolE. The impact on the rate of bacterial growth was not reported, so as an initial step, cell viability was measured over time at several compound concentrations. In the present study, a volume sufficient for determination of the optical density and cell numbers and for preparation of RNA was desired. A standard 20-ml-volume culture in LB was employed for all experiments, and compound 2 was added at various concentrations, using the MIC for this strain of 0.25 μg/ml as a baseline. As can be seen in Fig. 2A, concentrations above 0.6 μg/ml resulted in a significant decline in bacterial viability. The viability experiments were purposely performed at a bacterial mass that was necessary for the direct, rapid isolation of RNA from the cultures, without prior centrifugation or other manipulations to concentrate the cells. Compound 2 (at 0.3 and 1.2 μg/ml) was added to a culture of E. coli Δ*acrB* at an OD_600_ of 0.5 (5 × 10^8^ CFU/ml) for 30 min prior to RNA isolation by addition of a portion of the culture to hot acid phenol. These concentrations of the compound were just below and just above the levels where effects on growth were detected (Fig. 2A). The cultures were monitored after the 30-min period, and it was noted that the optical density declined at the higher compound concentrations over time (Fig. 2B).

### Transcriptional effects of Lol inhibition by RNA-seq.

The data for genes that were upregulated 5-fold or more by treatment with compound 2 are listed in Table 1; complete transcript-level data are presented in Tables S1 and S2 in the supplemental material. Comparative graphs derived from the transcriptome data for duplicate measurements and control cells versus compound-treated cells are shown in Fig. S2 in the supplemental material. Poorly expressed genes with very low numbers of reads per million (below 16) were removed from the data in the tables (22). An average level of upregulation of RNA in treated cells exceeding 10-fold the average level of regulation in control cells was observed for 38 genes with the lower concentration of the compound and 74 genes with the higher concentration of the compound. In almost all cases, a dose-response relationship in which higher compound concentrations resulted in greater magnitudes of RNA concentration changes could be seen. The majority of RNA species that were upregulated were for genes associated with envelope stress. Several different stress-induced regulons contributed to these changes. In the 30 min of compound 2 treatment, there was no increase in the levels of mRNA for the compound target, LolCDE. The levels of expression of all of the *lol* genes (*lolA*, *lolB*, and *lolCDE*) were also checked by qPCR (see below).

**TABLE 1:** Genes whose RNA was upregulated >5-fold in cells treated with the LolCDE inhibitor compared with the level of regulation in control cells

Gene^*a*^	Fold upregulation at compound 2 concn of^*b*^:		Product^*c*^	Predicted function(s) and/or comment(s)
	0.3 μg/ml	1.2 μg/ml		
*osmB*	114.75	194.64	Lipoprotein	Putative membrane protein, osmotic adaptation
*ycfJ*	102.84	172.38	Predicted protein	
*bdm*	88.28	138.58	Biofilm-dependent modulation protein	The RcsCDB His-Asp phosphorelay positively regulates Bdm (biofilm-dependent modulation)
*wcaE*	72.43	135.85	Predicted glycosyl transferase	Resistance to acid and to thermal stress
*wza*	46.80	93.05	Capsular polysaccharide translocon	Resistance to acid and to thermal stress
*wzb*	45.10	95.34	Protein-tyrosine phosphatase	Resistance to acid and to thermal stress
*ypeC*	41.97	59.57	Putative periplasmic protein	
*ypfG*	37.65	44.18	Predicted protein	Possible β-barrel structure on the basis of the sequence
*yaiY*	37.19	50.51	Predicted inner membrane protein	Protein involved in envelope, osmotic, and other stresses
*ygaC*	34.03	46.72	Predicted protein	Benzoate response, cytoplasmic pH stress response, coregulated with YgmABC
*spy*	31.52	60.63	Envelope stress-induced periplasmic protein	Stress-induced protein
*yjbJ*	28.22	57.10	Predicted stress response protein	Stress-induced protein
*ydeI*	28.06	66.02	Conserved protein	YdeI responds to hydrogen peroxide stress
*yghA*	25.59	57.66	Predicted glutathionylspermidine synthase, NAD(P)-binding Rossmann fold domain	Putative enzyme, not classified
*osmY*	25.29	52.53	Periplasmic protein	Osmotic adaptation
*ygdI*	25.16	53.38	Predicted protein	Lipoprotein induced by RpoS
*ymgG*	24.63	26.53	Predicted protein	*ymgG-ymgD* is a possible toxin-antitoxin system
*mliC*	24.44	36.51	Predicted lipoprotein	MliC protein inhibits the activity of c-type lysozyme
*yjbF*	24.28	45.69	Predicted lipoprotein	Expression of *yjbF* is positively regulated by RcsC
*ivy*	22.68	37.01	Inhibitor of vertebrate c-type lysozyme	Lysozyme inhibitor, protects peptidoglycan when the outer membrane is permeabilized
*ymgD*	19.43	24.26	Predicted protein	*ymgG-ymgD* is a possible toxin-antitoxin system
*ytjA*	18.30	35.85	Predicted protein	In operon with OsmY?
*ybhP*	17.60	33.10	Cytoplasmic	Function unknown
*wzc*	17.35	44.98	Protein-tyrosine kinase	Resistance to acid and to thermal stress
*yncJ*	16.53	33.64	Predicted protein	CpxA-regulated stress response
*yjbG*	15.59	30.16	Conserved protein	Extracellular polysaccharide
*yjbE*	15.38	32.49	Predicted protein	
*rcsA*	15.21	22.95	DNA-binding transcriptional activator; the coregulator is RcsB	Regulator of surface polysaccharides and antigens
*ybgS*	15.06	32.04	Conserved protein	Putative regulator, not classified
*yebE*	14.40	31.47	Conserved protein	YebE is an inner membrane protein with one predicted transmembrane domain
*cpxP*	12.57	20.10	Periplasmic protein that combats stress	Periplasmic space
*clsB*	12.32	24.47	Cardiolipin synthase 2	Stationary-phase cardiolipin synthase, phospholipids
*yegS*	12.15	18.37	Conserved protein	
*wcaF*	12.12	25.69	Predicted acyl transferase	Putative enzyme, resistance to desiccation, colanic acid biosynthesis (M antigen), resistance to acid and to thermal stress
*hslJ*	11.29	12.86	Heat-inducible protein	Heat shock protein, adaptations, atypical conditions
*yajI*	11.13	16.62	Predicted lipoprotein	
*ysaB*	11.11	11.23	Predicted protein	Uncharacterized lipoprotein
*wcaA*	11.06	25.98	Predicted glycosyl transferase	Resistance to desiccation, colanic acid biosynthesis (M antigen), resistance to acid and to thermal stress
*yjdP*	9.52	10.62	Conserved protein	Function unknown, putative signal peptide
*ycfT*	9.33	16.52	Predicted inner membrane protein	Adjacent to LolCDE, divergent transcript, controls biofilm formation
*gmd*	9.20	22.66	GDP–d-mannose dehydratase, NAD(P) binding	Sugar nucleotide biosynthesis, conversion, resistance to desiccation, resistance to acid and to thermal stress
*osmC*	8.91	16.55	Osmotically inducible, stress-inducible membrane protein	Osmotic adaptation
*dgcZ*	8.84	15.60		Diguanylate cyclase
*degP*	8.66	11.86	Serine endoprotease (protease Do), membrane associated	Predicted to be required for global protein degradation
*osmF*	8.55	15.28	Predicted periplasm-localized binding component of an ABC superfamily transporter	Putative transporter
*katE*	8.50	18.43	Hydroperoxidase HPII (catalase)	Enzyme, detoxification
*yhbO*	8.43	15.64	Predicted intracellular protease	*yhbO* mutant is highly sensitive to oxidative, thermal, UV, and pH stresses
*yaaX*	8.38	12.05	Predicted protein	RpoS stress induced
*osmE*	8.38	15.42	DNA-binding transcriptional activator	Regulator of global regulatory functions
*yqaE*	8.17	17.15	Predicted membrane protein	Stress response protein
*yfdC*	8.04	13.23	Predicted inner membrane protein	Putative transporter, not classified
*ygdR*	7.78	12.10	Predicted protein	Rcs induces the gene in response to cell wall damage (peptidoglycan)
*otsB*	7.77	14.52	Trehalose-6-phosphate phosphatase, biosynthetic	Enzyme, osmotic adaptation
*sra*	7.63	3.16	30S ribosomal subunit protein S22	Structural component of ribosomal proteins
*ybaY*	7.57	14.14	Predicted outer membrane lipoprotein	Lipoprotein with unknown function
*ydhS*	7.31	14.08	Conserved protein with FAD/NAD(P)-binding domain	Putative oxidoreductase
*ybdK*	7.18	15.12	Gamma-glutamyl:cysteine ligase	A weak gamma-glutamyl:cysteine ligase
*ybiO*	7.09	11.83	Predicted mechanosensitive channel	Putative transporter with unknown function
*ymdF*	7.02	15.25	Conserved protein	
*fbaB*	6.82	12.41	Fructose bisphosphate aldolase class I	
*ybdR*	6.78	12.11	Predicted oxidoreductase, Zn dependent and NAD(P) binding	Putative enzyme, not classified
*ecnB*	6.66	13.46	Cell envelope bacteriolytic lipoprotein	EcnAB form a linked toxin-antitoxin addiction module, entericidin A; antidote to lipoprotein entericidin B
*loiP*	6.56	9.69	Predicted peptidase	Enzyme, degradation of proteins, peptides, and glycopeptides
*yliI*	6.22	10.85	Predicted dehydrogenase	Putative enzyme, not classified
*rhsB*	6.08	8.99	*rhsB* element core protein RshB	Open reading frame with transposon-related functions
*yceB*	6.06	7.71	Predicted lipoprotein	Lipoprotein
*dppB*	5.95	11.38	Dipeptide transporter, membrane component of ABC superfamily	Transport, protein and peptide secretion
*tomB*	5.91	11.20	Toxin overexpression modulator	Induced during biofilm formation
*dsrA*	5.73	10.66	Regulatory, antisense RNA	Regulatory RNA, regulates transcriptional silencing by H-NS protein, enhances translation of RpoS antisense RNA
*ytfK*	5.70	9.04	Conserved protein	
*wcaC*	5.70	12.78	Predicted glycosyl transferase	Resistance to desiccation, colanic acid biosynthesis (M antigen), resistance to acid and to thermal stress
*yhhA*	5.59	9.91	Conserved protein	
*elaB*	5.56	10.14	Conserved protein	
*ydeJ*	5.55	8.26	Conserved protein	
*ydcT*	5.54	8.78	Spermidine/putrescine transporter	Putative transporter, not classified
*wcaG*	5.44	14.19		GDP-fucose synthase is a bifunctional enzyme, catalyzes the two-step synthesis of GDP-fucose
*ycaC*	5.41	10.17	Predicted hydrolase	
*ugd*	5.37	8.24	UDP-glucose 6-dehydrogenase	Converts UDP-glucose to UDP-glucuronic acid for, colanic acid biosynthesis
*raiA*	5.37	9.24	Cold shock protein associated with 30S ribosomal subunit	Putative regulator, not classified
*dppC*	5.20	11.03	Dipeptide transporter, membrane component of ABC superfamily	Transport, protein and peptide secretion
*yjbH*	5.16	9.76	Predicted porin	Overexpression of the *yjbEFGH* operon alters colony morphology
*dppD*	5.10	10.88	Dipeptide transporter, ATP-binding component of ABC superfamily	Transport, protein and peptide secretion
*ybjP*	5.07	7.64	Predicted lipoprotein	Putative enzyme, not classified
*yceJ*	5.06	5.57	Predicted cytochrome *b*_561_	Putative enzyme, not classified
*narK*	5.03	27.62	Nitrate/nitrite transporter	Transport of small molecules (anions)
*otsA*	4.93	8.81	Trehalose-6-phosphate synthase	Enzyme, osmotic adaptation
*sbp*	4.89	6.88	Sulfate transporter subunit, periplasm-localized binding component of the ABC superfamily	Transport of small molecules (anions)
*ymgE*	4.86	11.78	Predicted inner membrane protein	Transglycosylase-associated protein
*yiaG*	4.77	8.29	Predicted transcriptional regulator	
*ydcS*	4.70	8.04	Periplasm-localized binding component of an ABC superfamily predicted spermidine/putrescine transporter	Putative transporter, not classified
*poxB*	4.68	9.33	Pyruvate dehydrogenase (pyruvate oxidase), thiamine dependent, FAD binding	Enzyme, degradation of small molecules (carbon compounds)
*ycgB*	4.60	10.03	Conserved protein	
*yeaG*	4.56	9.52	Conserved protein with nucleoside triphosphate hydrolase domain	
*dxr*	4.48	6.16	1-Deoxy-d-xylulose 5-phosphate reductoisomerase	
*msyB*	4.45	8.10	Predicted protein	Protein and peptide secretion
*ascB*	4.44	6.50	Cryptic 6-phospho-beta-glucosidase	Enzyme, degradation of small molecules (carbon compounds)
*ybhN*	4.43	9.14	Conserved inner membrane protein	
*cysN*	4.42	8.40	Sulfate adenylyltransferase, subunit 1	Enzyme, central intermediary metabolism (sulfur metabolism)
*cysD*	4.38	6.56	Sulfate adenylyltransferase, subunit 2	Enzyme, central intermediary metabolism (sulfur metabolism)
*cysA*	4.27	6.83	Sulfate/thiosulfate transporter subunit, ATP-binding component of ABC superfamily	Transport of small molecules (anions)
*csiD*	4.26	6.39	Predicted protein	
*ybbA*	4.23	6.56	Predicted transporter subunit, ATP-binding component of ABC superfamily	Putative transporter, not classified
*dacC*	4.23	6.41	d-Alanyl–d-alanine carboxypeptidase (penicillin-binding protein 6a)	Enzyme, peptidoglycan
*ynfD*	4.19	6.85	Predicted protein	CpxA-regulated stress response
*ycaP*	4.17	7.00	Conserved inner membrane protein	
*ygaU*	4.13	8.01	Predicted protein	
*rcnB*	4.12	4.87		
*dps*	4.11	8.30	Fe-binding and storage protein	Regulator, global regulatory functions
*acrD*	4.10	6.31	Aminoglycoside/multidrug efflux system	Putative transporter, drug/analog sensitivity
*yodD*	4.09	6.76	Predicted protein	
*yceK*	4.09	8.00	Predicted lipoprotein	
*dppA*	4.08	6.27	Periplasm-localized binding component of an ABC superfamily dipeptide transporter	Transport, protein and peptide secretion
*blc*	3.99	8.17	Outer membrane lipoprotein (lipocalin)	Macromolecule synthesis, modification (lipoprotein)
*ddpF*	3.99	6.63	ATP-binding component of an ABC superfamily d-Ala–d-Ala transporter	
*ldtD*	3.95	7.72		
*yehX*	3.91	6.49	Predicted transporter subunit, ATP-binding component of ABC superfamily	Putative transporter, not classified
*yfcG*	3.90	7.27	Glutathione *S*-transferase	
*yebV*	3.89	6.18	Predicted protein	

a Gene designations are from the Escherichia coli BW25113 genome sequence.

b Fold upregulation over the control values at the two compound concentrations. All values are averages from two RNA-seq determinations for each condition with two biological replicates.

c Gene product from EcoGene (http://www.ecogene.org).

In terms of decreased levels of RNA in response to pharmacological inhibition of lipoprotein transport, the number of genes for which major reductions in RNA levels were seen was much smaller than the number for which increases in RNA levels were seen. As can be seen in Table 2, the vast majority of genes with reduced levels of RNA expression encode flagellar components. This may be the consequence of disruption of flagellar assembly due to mislocalization of the lipoprotein FlgH, the L-ring subunit of the flagellar basal body. Consequently, failure to assemble the hook/basal body complex and retain the anti-sigma factor FlgM in the cytoplasm would lead to the global repression of flagellar gene expression (23, 24).

**TABLE 2:** Genes whose RNA was downregulated >5-fold in cells treated with the LolCDE inhibitor compared with the level of regulation in control cells

Gene^*a*^	Fold downregulation at compound 2 concn of^*b*^:		Product^*c*^	Predicted function
	0.3 μg/ml	1.2 μg/ml		
*flhD*	−4.17	−11.89	DNA-binding transcriptional dual regulator with FlhC	Regulator, surface structures
*fliE*	−4.11	−15.86	Flagellar basal body component	Structural component, surface structures
*fliF*	−4.03	−21.47	Flagellar basal body MS ring and collar protein	Structural component, surface structures
*flhC*	−3.84	−13.37	DNA-binding transcriptional dual regulator with FlhD	Regulator, surface structures
*intG*	−3.81	−25.97	Predicted defective phage integrase (pseudogene)	
*fliG*	−3.76	−16.01	Flagellar motor switching and energizing component	Structural component, surface structures
*fliH*	−3.48	−14.28	Flagellar biosynthesis protein	Transport, surface structures
*fliI*	−3.35	−12.52	Flagellum-specific ATP synthase	Enzyme, surface structures
*fliJ*	−3.29	−9.77	Flagellar export apparatus chaperone	Structural component, surface structures
*ompF*	−3.19	−12.49	Outer membrane porin 1a (Ia)	Membrane, outer membrane constituents
*fliA*	−3.16	−7.68	RNA polymerase, sigma 28 (σF) factor	σ factor, surface structures
*yciX*	−3.16	−12.44	Predicted protein	
*flgC*	−3.10	−6.25	Flagellar component of cell-proximal portion of basal body rod	Structural component, surface structures
*fliK*	−3.08	−8.02	Flagellar hook-length control protein	Structural component, surface structures
*yecR*	−3.04	−9.26	Predicted protein	

a Gene designations are from the Escherichia coli BW25113 genome sequence.

b Fold downregulation over the control values at the two compound concentrations. All values are averages from two RNA-seq determinations for each condition with two biological replicates.

c Gene product from EcoGene (http://www.ecogene.org).

### Transcriptional impact of antibiotic inhibition on select genes.

**FIG 3:**
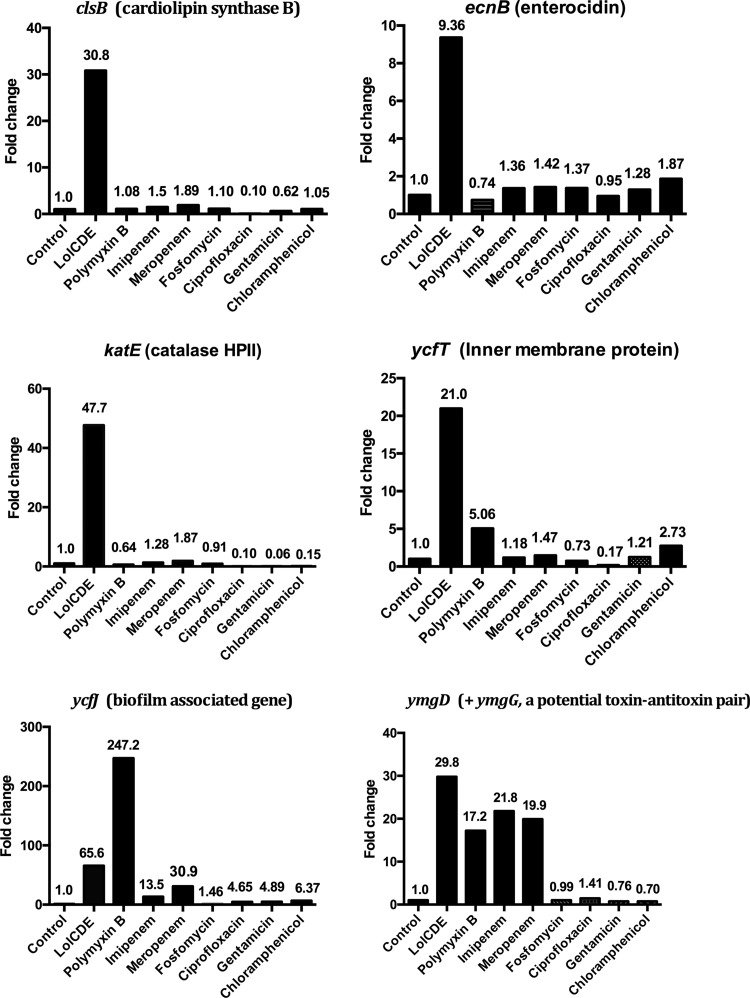

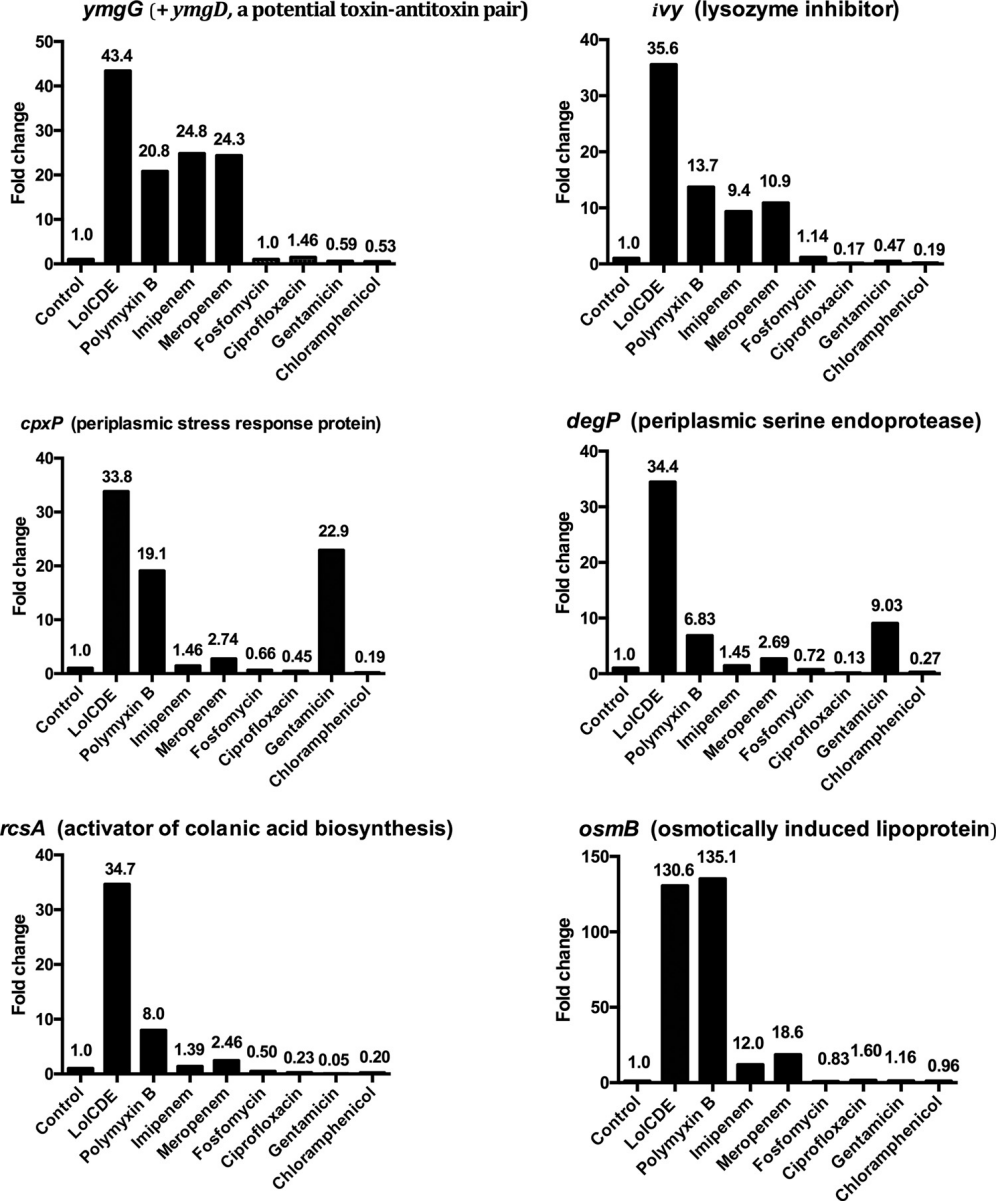

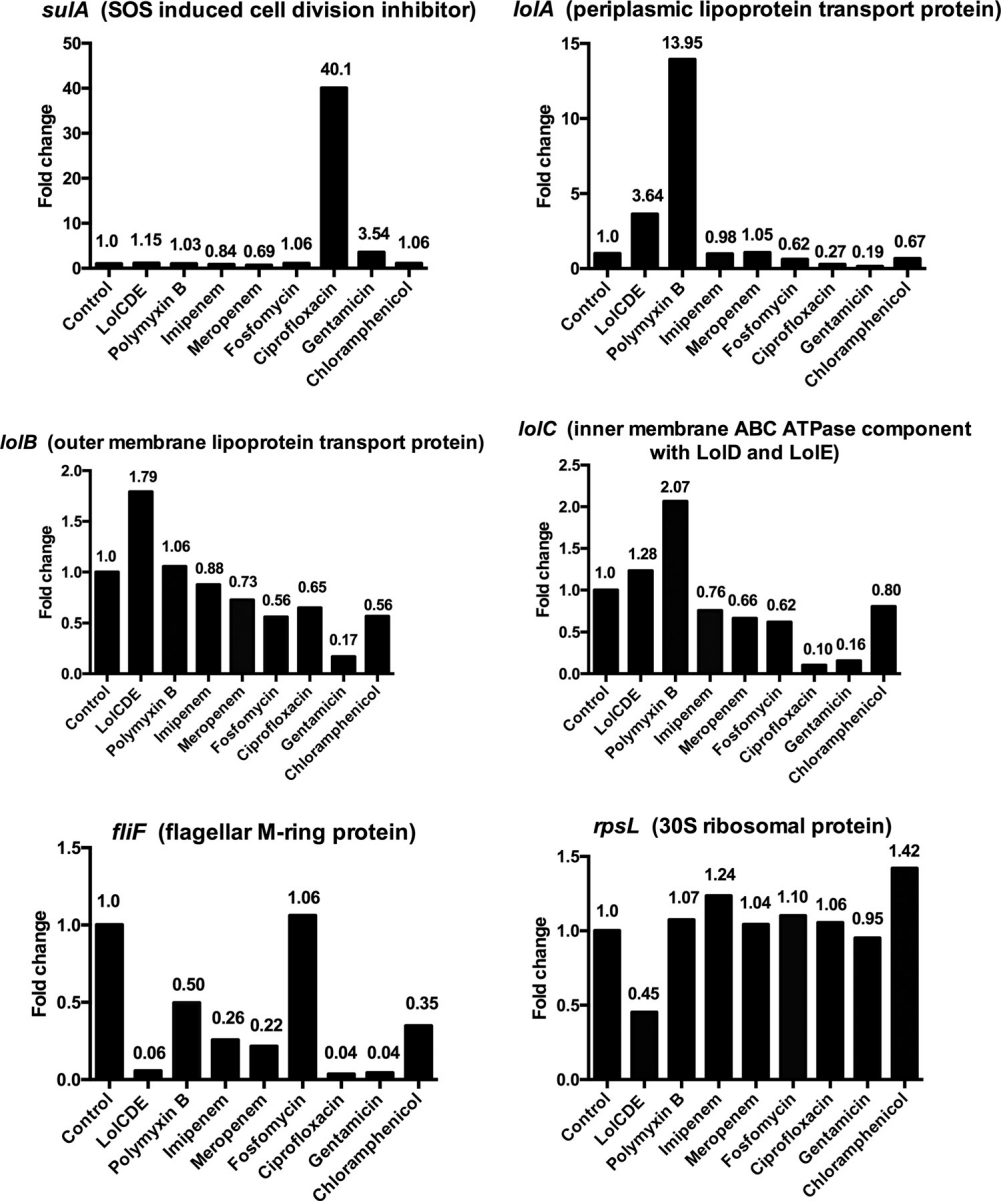
Quantitative PCR of RNA extracted from cells treated with compound at a concentration sufficient to inhibit their growth (see Fig. S1 in the supplemental material) when it was added to cultures when they were at an OD_600_ of 0.5 (the conditions employed for RNA extraction). RNA was extracted from 20-ml cultures that had been treated for 30 min with the indicated antibiotics. cDNA was prepared from the RNA as described in Materials and Methods. Values are for duplicate samples obtained on different days and were calculated from the results of qPCR. Values represent the levels of expression compared to the level for untreated cells (for which the level of expression was set equal to 1.00). Compounds were added to the cultures when the cells were at an OD_600_ of 0.5. Compound concentrations were 1.2 μg/ml for compound 2 (the LolCDE inhibitor), 4 μg/ml for polymyxin B, 1 μg/ml for imipenem, 2 μg/ml for meropenem, 0.25 μg/ml for fosfomycin, 1 μg/ml for ciprofloxacin, 16 μg/ml for gentamicin, and 1 μg/ml for chloramphenicol.

Next we selected several genes that demonstrated significant upregulation or downregulation and determined the concentrations of mRNA for those genes by quantitative PCR (qPCR). Their transcript levels were also compared to those obtained following treatment of cells with seven other antibiotics with several distinct mechanisms of action, in order to determine if a subset of the genes under study was uniquely regulated by the LolCDE inhibitor or whether these were also affected by antibiotic disruption of other cell physiological properties. Preliminary experiments with high-volume shaking cultures established the minimal concentrations of the various antibiotics that were inhibitory to E. coli Δ*acrB* growth under conditions analogous to those employed in the RNA-seq experiments with the higher cell numbers and higher concentrations of the LolCDE inhibitor. The E. coli strain was exposed to these compounds at concentrations 2 times the inhibitory levels (along with the LolCDE compound at the higher concentration) for 30 min, conditions identical to those used in the RNA-seq experiments. RNA was extracted, purified, and converted to cDNA for qPCR determinations. Results for individual selected genes are presented in Fig. 3.

We used *rpsL*, encoding the ribosomal protein S12, as a housekeeping gene whose level of transcription should not vary significantly from that for the control cells to determine the efficiency and variability of the RNA extractions in the different experiment using cells treated with the LolCDE inhibitor or various antibiotics. All of the *rpsL* transcript levels were within ∼2-fold of those for the control cells (Fig. 3). In the presence of the LolCDE inhibitor, several genes associated with cell envelope stress were markedly upregulated, and this result was confirmed by qPCR. The concentrations of mRNA for the genes *clsB*, *katE*, *ycfT*, and *ecnB* were reproducibly elevated in bacteria treated with the LolCDE inhibitor, and the levels were very similar to the levels observed in the RNA-seq experiments. None of the genes in this group showed alterations in their transcript levels when the bacteria were treated with several other antibiotics. The largest increase in the level of expression of mRNA resulting from LolCDE inhibition was seen for *osmB*, and this result was confirmed by qPCR. The levels of *osmB m*RNA were also markedly elevated by polymyxin B treatment. The expression levels of *yegS* were also elevated by treatment with the LolCDE inhibitor, and additional modest levels of increase were noted with the two β-lactam compounds (imipenem and meropenem), as well as with polymyxin B. A similar pattern emerged with the hyperosmosis-associated gene *ycfJ*, whose transcript levels were notably higher following treatment with the β-lactams and polymyxin B and were similar to the levels observed with the LolCDE inhibitor. The levels of the mRNAs for the putative toxin-antitoxin pair *ymgD* and *ymgG* were also elevated in the presence of the LolCDE inhibitor, the two β-lactam compounds, and polymyxin B. A similar pattern in response to antibiotic treatment was observed for *ivy*, which protects cells permeabilized by chemical or physical stresses (25). The transcript levels for CpxP, a protein involved in the regulation of degradation of misfolded proteins, were also increased by the LolCDE inhibitor, polymyxin B, and gentamicin. Similarly, transcripts specifying DegP, which functions in the same degradation pathway as CpxP, were also increased by these treatments, albeit to a lesser extent. Little effect on any of the LolCDE inhibitor-induced mRNA changes was observed following ciprofloxacin exposure; however, when the effect of ciprofloxacin exposure on the SOS gene *sulA* was tested, it was found to be upregulated by this antibiotic, as expected (26).

Among the genes whose mRNAs showed a decrease when lipoprotein transport to the outer membrane was blocked, one (*fliF*) was examined by qPCR. In addition to being affected by the LolCDE inhibitor, its concentration was also reduced by treatment with the other antibiotics tested, with the exception of fosfomycin, the inhibitor of MurA, an enzyme involved in the early stages of the peptidoglycan biosynthetic pathway. It appears that a number of different cellular perturbations have the ability to affect the highly orchestrated and hierarchical regulation of flagellar genes.

### Comparison of gene expression in the parent strain and resistant mutants.

**FIG 4:**
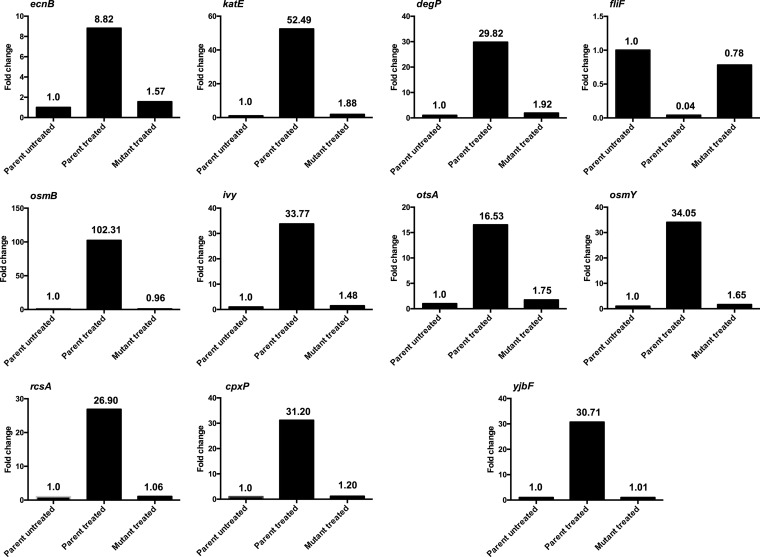
Quantitative PCR of untreated parental strain E. coli BW25113 Δ*acrB*, the parental strain exposed to compound 2 at 1.2 μg/ml, and the E. coli BW25113 Δ*acrB* LolC N256K mutant exposed to compound 2 at 1.2 μg/ml. The levels of transcription of key genes in each of the three regulatory pathways (indicated after the gene) affected by the compound were measured. Transcript levels were determined for *ecnB* (σ^S^), *katE* (σ^S^), *osmB* (σ^S^ and Rcs), *ivy* (Rcs), *rcsA* (CpxA/R), *cpxP* (CpxA/R), *degP* (CpxA/R), *fliF* (flagellum synthesis), *otsA* (σ^S^), *osmY* (σ^S^), and *yjbF* (Rcs). Values are averages for two biological replicates and were calculated from the results of qPCR. Values represent the levels of expression compared to that for untreated cells (for which the level of expression was set equal to 1.00).

One possibility is that the observed changes in the levels of expression of the stress response genes are not mediated directly by LolCDE inhibition but instead are indirect effects of the compound on the regulatory pathways. To address this possibility, a previously reported compound 2-resistant mutant (19, 27) with a mutation in LolC (N256K) was isolated, and duplicate cultures of the mutant and parent strain were treated with the compound (1.2 μg/ml) for 30 min. Duplicate cultures of untreated samples were also tested. RNA was extracted from all six cultures and processed for qPCR as described above. The levels of expression of selected upregulated genes involved in each of the stress pathways, as well as those of the downregulated flagellar genes, were assessed. As can be seen in Fig. 4, the mutant with the LolC mutation resistant to the compound no longer exhibited upregulation of the representative genes involved in the three stress pathways, nor was flagellar gene expression downregulated in this strain. In contrast, the treated parent strain exhibited changes in the levels of expression of the selected genes.

## DISCUSSION

Earlier work (19) established that the compound used in the present study, compound 2, stimulated peptidoglycan damage, resulting in activation of the *ampC* promoter, and also led to inhibition of the lipoprotein outer membrane transport pathway in E. coli, among other effects. Mutants resistant to the action of the compound were selected, and amino acid changes associated with resistance were found to reside in two subunits of the LolCDE complex. Therefore, LolC and LolE likely represent the direct targets of the Lol pathway inhibitor. These mutations individually resulted in a large increase in the MIC of the compound and led to cross-resistance to another small molecule also recently identified to be a LolCDE inhibitor (27). Our goal in the present study was to characterize the immediate transcriptional responses of E. coli to inhibition of LolCDE by the compound. This was accomplished by performing RNA-seq analysis with two different concentrations of the compound, a concentration just below and a concentration just above the concentration that affected cell growth (measured by determination of both the OD_600_ and the number of CFU per milliliter). These results were compared to those obtained with control cells, which received no compound. While RNA-seq can measure only transcript levels, the compound and antibiotics tested likely directly or indirectly affected the transcriptional regulation of particular genes; however, we cannot exclude the possibility that the effect was posttranscriptional at the level of mRNA stability.

Treatment with the LolCDE inhibitor led to a loss of bacterial viability and cell lysis, similar to the results seen previously following LolCDE depletion (28). No increase in the level of the transcripts of genes encoding the subunits of the actual target of compound 2, the LolCDE complex, was seen when E. coli was treated with the compound. There was, however, a modest 3.5-fold increase in *lolA* transcript levels at the higher concentration of the inhibitor, a result also seen in past experiments employing LolCDE depletion (28). Very large changes in the concentrations of mRNA for many genes that respond to periplasmic and cell envelope stress were observed following treatment with compound 2. In the case of chemical inhibition of the LolCDE function, the stress responses (29, 30) could be predominantly due to signaling controlled by three regulatory systems: the two-component histidine kinase and response regulators CpxA/R (31–33), the three-component Rcs system (34–37), and *rpoS* (σ^S^)-mediated stress responses (38–41). The extracytoplasmic response is controlled by at least two partially overlapping signal transduction systems, the Cpx two-component system and the σ^E^-mediated system. Neither *rpoE*-associated (42–44) nor *rpoH*-associated (45, 46) genes were upregulated by the LolCDE inhibitor compound under the conditions tested. The 30-min exposure to the inhibitor (approximately 1.5 cell generations) left the transcript levels of the σ^E^-induced β-barrel pathway chaperone genes *skp*, *fkpA*, and *sur*, as well as the levels of RNA for several known *rpoE* regulon genes, such as *bamD*, *bamB*, *rpoD*, *dsbC*, *yeaY*, and *yaeI*, unchanged (43, 47). Surprisingly, although the β-barrel system includes several lipoproteins (BamB and the essential lipoprotein BamD) associated with its function, no changes in the levels of their respective mRNAs or the levels of the transcripts of genes encoding other lipoproteins of the Bam pathway, BamC or BamE, were observed (3, 6). Longer exposure to the compound and prolonged inhibition may be necessary to elicit changes. The *rpoH* (σ^32^)-controlled genes are primarily induced by protein misfolding in association with heat shock. The genes known to be controlled by σ^32^ include *dnaK*, *dnaJ*, and *grpE*, the mRNAs for which also do not show altered levels during LolCDE inhibition (46). Likewise, no effect on *mdtA*, *mdtB*, *mdtC*, *mdtD*, or *tolC*, associated with the BaeSR system, was observed (48).

Among the genes induced in response to the LolCDE inhibitor compound were *yncJ*, *yqaE* and *cpxP*, as well as *spy*, which encodes the periplasmic chaperone and which is regulated by the CpxA/R stress response (32, 33, 49, 50). The general stress response sigma factor σ^S^ regulates *osmY* (encoding a periplasmic stress protein [51, 52]), *poxB*, *ydcS*, and *katE*, and these were among the genes for which the mRNA levels were elevated by exposure of cells to the LolCDE inhibitor (39). Other σ^S^ stress-related protein-encoding genes whose RNA levels were observed to be increased were *otsA* and *otsB*, whose gene products synthesize trehalose in response to osmotic stress (41, 53). The levels of expression of RNA for a number of genes associated with the Rcs phosphorelay system, including *osmB*, the Rcs regulator *rcsA*, and the genes for the colanic acid biosynthetic pathway, including *wcaA*, *wcaB*, *wcaC*, *wcaD*, *wcaE*, *wza*, *wzb*, and *wzc* (54–57), were also found to be increased. Also upregulated by the Rcs system was *ivy*, which protects peptidoglycan when the outer membrane is permeabilized (58).

Several of the RNAs responding to lipoprotein transport inhibition encode lipoproteins: the previously mentioned OsmB, an osmotic stress protein whose expression is controlled by both σ^S^ and Rcs (54). Other lipoproteins include genes encoding OsmC (a stress-induced lipoprotein), YgdL, MliC, YajL, YbaY, YceB, YbjP, and YjbF (a lipoprotein also regulated by Rcs) (34, 35, 37). Therefore, the commonality of all of these genes is their response to signaling pathways via regulators sensing periplasmic misfolding or perturbation of the outer membrane caused by a defect in lipoprotein trafficking.

Many fewer large-magnitude changes in the levels of expression genes showing decreases in their transcript levels following interference with lipoprotein trafficking were found. The levels of expression of virtually the entire set of mRNAs for genes associated with flagellum formation were markedly lower, and these genes comprised the group with the largest negative changes in expression (59, 60). This could be explained as a direct consequence of failure to assemble the flagellar ring due to mislocalization of the lipoprotein FlgH, which depends on the Lol pathway for its outer membrane targeting. Other genes whose transcript levels were also lower included *ompF*, previously shown to be controlled by CpxA/R (61). It is unclear why *ompF* responds differently from other CpxA/R-regulated genes that are adversely affected by LolCDE inhibition.

To determine if any of the responses observed were also induced by exposure to other antibiotics with different mechanisms of action, bacteria were exposed to several different antibiotics, and their mRNAs were analyzed by qPCR. Compared to a group of selected genes from the RNA-seq experiment, it was found that the transcript levels for four of the genes tested were elevated only by exposure to the LolCDE inhibitor. One of these genes was *ecnB*, coding for the bacteriolytic toxin part of a toxin-antitoxin pair which has been implicated in σ^S^-regulated osmolarity stress and bacteriolysis (62, 63). Interestingly, the gene for the companion antitoxin, *ecnA*, was not upregulated in this case; this should lead to cell death. Another gene was *katE*, which encodes a hydroperoxidase also regulated by σ^S^ and which is induced in response to oxidative stress (64, 65) The *clsB* gene, which encodes one of three cardiolipin synthases in E. coli (66), and *ycfT*, which encodes an inner membrane protein implicated in biofilm control and which is located immediately adjacent to and divergent from the genes encoding LolCDE, were also highly responsive to LolCDE inhibition. Among the group of antibiotics tested by qPCR at the inhibitory concentrations determined in this study, only the LolCDE inhibitor had an impact on the transcript levels of these four genes, although the level of transcription of *ycfT* mRNA also displayed a modest elevation with polymyxin B. The apparent specificity of these responses to pharmacological inhibition of LolCDE merits further future characterization of the genes for these proteins as possible candidates for use in cell-based reporter screens to find additional Lol pathway inhibitors. Other tested genes, such as *ycfJ*, implicated in biofilm formation, were also affected by polymyxin B and, to a lesser extent, by the two β-lactam antibiotics imipenem and meropenem, all of which have impacts on the bacterial cell envelope. The levels of both *ymgD* and *ymgG* mRNAs were elevated by the same compounds as well. The same was true of the expression of *ivy*, encoding another periplasmic protein that acts to protect peptidoglycan from hydrolysis by lysozyme and is induced by outer membrane permeabilization (67).

The question as to whether the responses are a direct result of LolCDE inhibition by the compound was addressed by isolating a resistant LolC mutant with the N256K mutation and repeating the compound exposure at the higher concentration employed for RNA-seq and qPCR with this mutant and identically treated parental strain E. coli BW25113 Δ*acrB*. The results obtained with cells of these strains were compared with those obtained with cells of the untreated parent. Quantitative PCR of representative genes involved in the different pathways clearly demonstrated that the transcript expression effects observed were through the inhibition of LolCDE by compound 2.

The purpose of this study was to examine the responses of E. coli to treatment with a compound that inhibits the function of LolCDE. Direct inhibition of LolCDE function led to a loss of cell viability and, ultimately, cell lysis. The blockade of lipoprotein transport was found to lead to compound dose-dependent levels of induction of selected cell stress pathways. Many of these are envelope stress responses that have previously been observed under various cell stimuli, such as disturbances in metabolism, periplasmic protein misfolding, or overproduction of lipoprotein NlpE (32, 68). Of interest, a small subset of the RNAs induced in response to LolCDE inhibition at the inhibitory concentrations tested in this study appeared to be unique rather than general responses to antibiotics with different mechanisms of action. These responses could be further defined for the design of refined cell-based reporter screens for Lol pathway inhibitors.

## Supplementary Material

Supplemental material
