# Supplementary material for: Transcriptional Responses of Escherichia coli to a Small-Molecule Inhibitor of LolCDE, an Essential Component of the Lipoprotein Transport Pathway
Source: J Bacteriol. 2016 Nov 4;198(23):3162–75. doi: 10.1128/JB.00502-16 (PMC5105897; doi:10.1128/JB.00502-16)
Supplement: Supplemental material [file supp_198_23_3162__index.html]

Transcriptional Responses of Escherichia coli to a Small-Molecule Inhibitor of LolCDE, an Essential Component of the Lipoprotein Transport Pathway — Supplemental material 

# Transcriptional Responses of Escherichia coli to a Small-Molecule Inhibitor of LolCDE, an Essential Component of the Lipoprotein Transport Pathway

## Supplemental material

- Supplemental file 1 -

  Fig. S1, growth curves for *E. coli* BW21135 with antibiotic compounds

  Fig. S2, scatter plots with RNA-seq results

  PDF, 386K
- Supplemental file 2 -

  Table S1, RNA-seq data for no compound versus 0.3 μg/ml LolCDE inhibitor

  XLSX, 296K
- Supplemental file 3 -

  Table S2, RNA-seq data for no compound versus 1.2 μg/ml LolCDE inhibitor

  XLSX, 323K
