# Supplementary material for: Transcriptional Responses of Escherichia coli to a Small-Molecule Inhibitor of LolCDE, an Essential Component of the Lipoprotein Transport Pathway
Source: J Bacteriol. 2016 Nov 4;198(23):3162–75. doi: 10.1128/JB.00502-16 (PMC5105897; doi:10.1128/JB.00502-16)

Transcriptional responses of *Escherichia coli* to a small molecule inhibitor of LolCDE, an essential component of the lipoprotein transport pathway

Supplemental Figures Legends

Supplemental Fig. 1. Growth curves of *E. coli* BW 21135 in key concentrations of antibiotic compounds. Growth was measured as optical density at 600 nm over a period of 5 hours. Compounds were added at approximately 0.5 OD to match the concentration of cells at the beginning of the 30 minute exposure period prior to RNA extraction.

Supplemental Fig. 2 . 2A. Control A & B values of the duplicate cell culture RNA-seq results plotted against each other. Scatter is minimal except at very low number of reads. 2B. LolCDE inhibitor duplicates plotted against each other. Scatter is minimal except at the lower number of reads. 2C. Plot of the control vs. the lower concentration of LolCDE inhibitor. Scatter has increased due to the differential expression of RNA as a result of the inhibitor. 2D. Plot of control vs. the higher concentration of LolCDE inhibitor. Scatter is markedly increased due to differential RNA expression.

Supplemental Figure 1

Growth	OD 600nm						
			0	2	3	4	5
control	ug/ml		0.474	2.309	2.974	3.563	4.161
Polymyxin b 4 μg/ml		4	0.474	0.193	0.316	0.105	0.081
Polymyxin b 2μg/ml		2	0.474	1.944	2.543	2.745	3.175
Polymyxin b		1	0.474	2.013	2.502	2.903	3.466

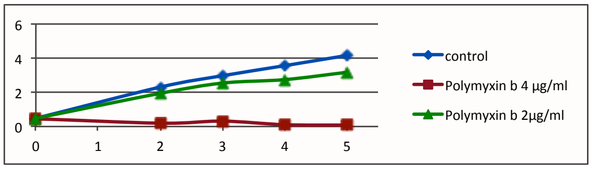

			0	2	3	4	5
control	ug/ml		0.474	2.309	2.974	3.563	4.161
Imipenem 1 μg/ml		1	0.474	0.66	0.488	0.275	0.201
Imipenem 0.5 μg/ml		0.5	0.474	1.975	2.255	2.694	3.172

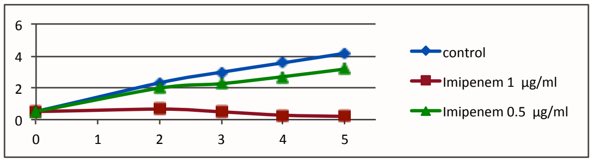

	Time (hours)		0	2	3	4	5
control			0.474	2.309	2.974	3.563	4.161
Meropenem 2 μg/ml		2	0.474	0.11	0.044	0.033	0.04
Meropenem 0.5 μg/ml		0.5	0.474	2.247	2.495	2.782	3.028

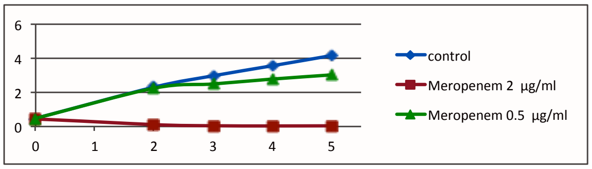

	Time (hours)		0	2	3	4	5
control			0.474	2.309	2.974	3.563	4.161
Fosfomycin 1 μg/ml		1	0.474	0.132	0.077	0.117	0.083
Fosfomycin 0.25 μg/ml		0.25	0.474	0.245	0.121	0.103	0.121

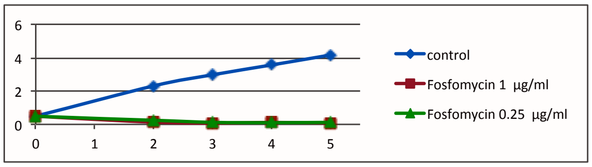

	Time (hours)		0	2	3	4	5
control			0.474	2.309	2.974	3.563	4.161
Ciprofloxacin 1 μg/ml		1	0.474	0.756	0.818	0.739	0.619
Ciprofloxacin 0.125 μg/ml		0.125	0.474	2.122	2.529	2.424	2.336

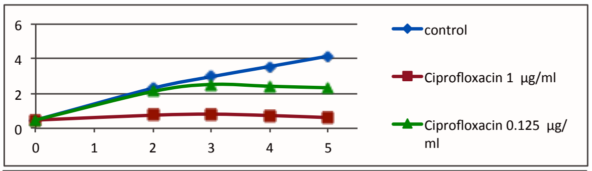

	Time (hours)		0	2	3	4	5
control			0.474	2.309	2.974	3.563	4.161
Gentamicin 16 μg/ml		16	0.474	0.522	0.442	0.37	0.356
Gentamicin 2 μg/ml		2	0.474	2.152	2.633	2.882	3.247

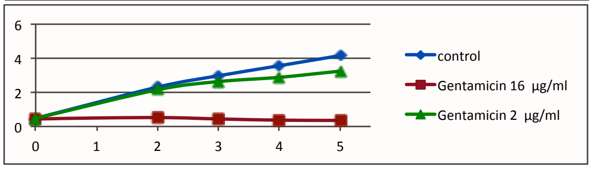

	Time (hours)		0	2	3	4	5
control		0	0.474	2.309	2.974	3.563	4.161
Chloramphenicol 1 μg/ml		1	0.474	1.141	1.315	1.392	1.527
Chloramphenicol 0.125 μg/		0.125	0.474	1.675	2.024	2.315	2.796

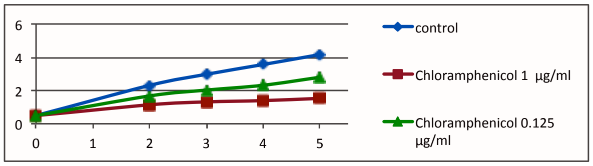

Supplemental Figure 2.

A. control A vs B

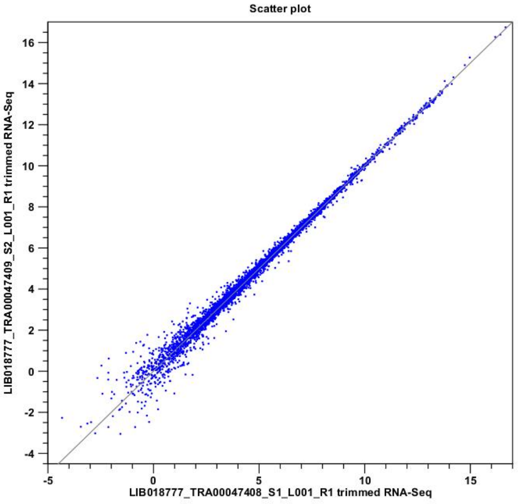

B. LolCDE inhibitor A vs B

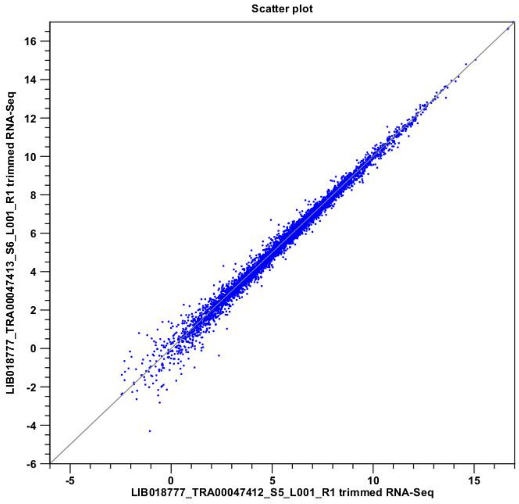

C. control vs LolCDE inh. (0.5 x MIC)

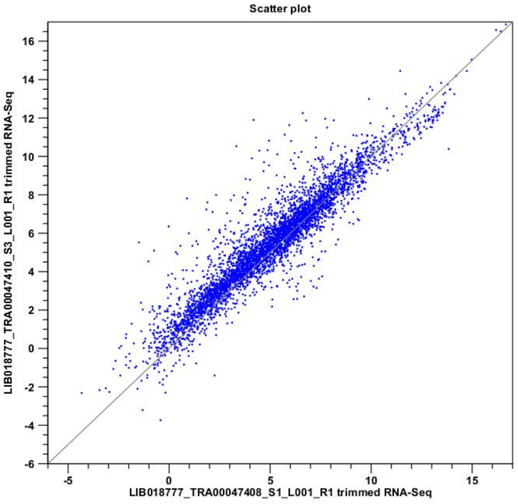

D. control vs LolCDE inh. (2x MIC)

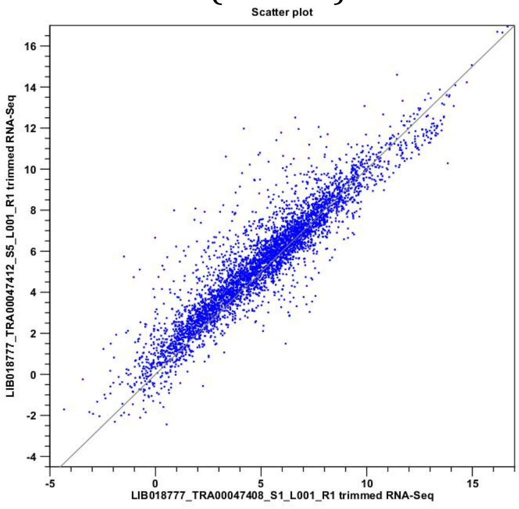

Supplement: Supplemental material [file JB.00502-16_zjb999094243so1.pdf]
